# Filamin A regulates EGFR/ERK/Akt signaling and affects colorectal cancer cell growth and migration

**DOI:** 10.3892/mmr.2022.12770

**Published:** 2022-06-14

**Authors:** Kun Wang, Tie-Nian Zhu, Rui-Jing Zhao

Mol Med Rep 20: 3671–3678, 2019; DOI: 10.3892/mmr.2019.10622

Following the publication of this article, an interested reader drew to the authors’ attention that various panels in the scratch-wound assays shown in Fig. 1C appeared to contain overlapping sections, such that the data may have been derived from a more limited selection of original sources where the data were intended to show the data from discrete experiments. Furthermore, the results shown in the Kaplan-Meier overall survival analysis plots in Fig. 4C appeared to be inconsistent with the date of publication of this article.

Independently, the Editorial Office also investigated the issues of concern in this article, and although the authors attenpted to explain these issues and requested the publication of a corrigendum, the Editor of *Molecular Medicine Reports* has declined this request and determined that this article should be retracted from the journal on the basis of an overall lack of confidence in the presented data. Upon receiving this decision from the Editor, the authors were not in agreement that the article should be retracted. The Editor apologizes to the readership of the Journal for any inconvenience caused.

